# Association of Global DNA Methylation and Global DNA Hydroxymethylation with Metals and Other Exposures in Human Blood DNA Samples

**DOI:** 10.1289/ehp.1306674

**Published:** 2014-04-25

**Authors:** Maria Tellez-Plaza, Wan-yee Tang, Yan Shang, Jason G. Umans, Kevin A. Francesconi, Walter Goessler, Marta Ledesma, Montserrat Leon, Martin Laclaustra, Jonathan Pollak, Eliseo Guallar, Shelley A. Cole, M. Dani Fallin, Ana Navas-Acien

**Affiliations:** 1 Department of Epidemiology,; 2 Welch Center for Prevention, Epidemiology and Clinical Research, and; 3 Department of Environmental Health Sciences, Johns Hopkins Bloomberg School of Public Health, Baltimore, Maryland, USA; 4 Fundacion de Investigacion del Hospital Clinico de Valencia-INCLIVA, Valencia, Spain; 5 Department of Respiratory Disease, Changhai Hospital, Second Military Medical University, Shanghai, China; 6 MedStar Health Research Institute, Hyattsville, Maryland, USA; 7 Georgetown-Howard Universities Center for Clinical and Translational Science, Washington, DC, USA; 8 Institute of Chemistry–Analytical Chemistry, Karl-Franzens University, Graz, Austria; 9 Aragon Health Sciences Institute, Zaragoza, Spain; 10 Department of Epidemiology, Atherothrombosis and Imaging, National Center of Cardiovascular Research-CNIC, Madrid, Spain; 11 Department of Medicine, Johns Hopkins Medical Institutions, Baltimore, Maryland, USA; 12 Department of Genetics, Texas Biomedical Research Institute, San Antonio, Texas, USA

## Abstract

Background: The association between human blood DNA global methylation and global hydroxymethylation has not been evaluated in population-based studies. No studies have evaluated environmental determinants of global DNA hydroxymethylation, including exposure to metals.

Objective: We evaluated the association between global DNA methylation and global DNA hydroxymethylation in 48 Strong Heart Study participants for which selected metals had been measured in urine at baseline and DNA was available from 1989–1991 (visit 1) and 1998–1999 (visit 3).

Methods: We measured the percentage of 5-methylcytosine (5-mC) and 5-hydroxymethylcytosine (5-hmC) in samples using capture and detection antibodies followed by colorimetric quantification. We explored the association of participant characteristics (i.e., age, adiposity, smoking, and metal exposure) with both global DNA methylation and global DNA hydroxymethylation.

Results: The Spearman’s correlation coefficient for 5-mC and 5-hmC levels was 0.32 (*p* = 0.03) at visit 1 and 0.54 (*p* < 0.001) at visit 3. Trends for both epigenetic modifications were consistent across potential determinants. In cross-sectional analyses, the odds ratios of methylated and hydroxymethylated DNA were 1.56 (95% CI: 0.95, 2.57) and 1.76 (95% CI: 1.07, 2.88), respectively, for the comparison of participants above and below the median percentage of dimethylarsinate. The corresponding odds ratios were 1.64 (95% CI: 1.02, 2.65) and 1.16 (95% CI: 0.70, 1.94), respectively, for the comparison of participants above and below the median cadmium level. Arsenic exposure and metabolism were consistently associated with both epigenetic markers in cross-sectional and prospective analyses. The positive correlation of 5-mC and 5-hmC levels was confirmed in an independent study population.

Conclusions: Our findings support that both epigenetic measures are related at the population level. The consistent trends in the associations between these two epigenetic modifications and the characteristics evaluated, especially arsenic exposure and metabolism, suggest the need for understanding which of the two measures is a better biomarker for environmental epigenetic effects in future large-scale epidemiologic studies.

Citation: Tellez-Plaza M, Tang WY, Shang Y, Umans JG, Francesconi KA, Goessler W, Ledesma M, Leon M, Laclaustra M, Pollak J, Guallar E, Cole SA, Fallin MD, Navas-Acien A. 2014. Association of global DNA methylation and global DNA hydroxymethylation with metals and other exposures in human blood DNA samples. Environ Health Perspect 122:946–954; http://dx.doi.org/10.1289/ehp.1306674

## Introduction

DNA 5-methylcytosine (5-mC) modifications are increasingly recognized as a key process in the pathogenesis of complex disorders, including cancer, diabetes, and cardiovascular disease (Feinberg 2010; Ordovas and Smith 2010). Several recent studies have investigated epigenetic functions of 5-hydroxymethylcytosine (5-hmC), a hydroxylated and methylated form of cytosine. The conversion of 5-mC to 5-hmC is a step prior to demethylation (Shen et al. 2013; Song et al. 2013) and seems to play a direct role in the regulation of gene expression (Branco et al. 2012). Although 5-hmC is able to bind to DNA methylation binding domain (MBD) 3 (Baubec et al. 2013), it reduces the binding of other MBD proteins to methylated DNA and prevents DNA methyltransferase (DNMT)-mediated methylation of the target cytosine (Tahiliani et al. 2009). Several studies have evaluated blood cell global DNA methylation and its determinants in population-based studies (reviewed by Terry et al. 2011). However, few human studies have evaluated global DNA hydroxymethylation. Specifically, the association between human blood cell global DNA methylation and global DNA hydroxymethylation has not been previously evaluated in epidemiologic studies.

Environmental exposures, such as to arsenic or cadmium, can disrupt gene expression (Andrew et al. 2008; Bourdonnay et al. 2009; Castillo et al. 2012; Cheng et al. 2012; Hossain et al. 2012; Su et al. 2006). Findings suggest that the health effects of environmental exposures, including exposure to metals, could be mediated in part by epigenetic mechanisms (Arita and Costa 2009; Reichard and Puga 2010; Ren et al. 2011; Smeester et al. 2011). Results of studies conducted *in vitro* and in primary human tissue samples also support that metals can have epigenetic effects (Huang et al. 2008; Kile et al. 2012; Lambrou et al. 2012; Smeester et al. 2011). No studies have evaluated environmental determinants of global DNA hydroxymethylation, including exposure to metals.

In this study, we examined the association between global DNA methylation and global DNA hydroxymethylation in a subsample of Strong Heart Study (SHS) participants who had metals measured in urine and also had buffy coat and blood available for DNA isolation (Lee et al. 1990; Scheer et al. 2012). In addition, we explored the association of participant characteristics (age, sex, education, adiposity, smoking, alcohol intake, metal exposure, and arsenic metabolism) with both global DNA methylation and global DNA hydroxymethylation. We had no *a priori* hypothesis on the direction of the associations under study.

## Methods

*Study population*. The SHS is a population-based cohort study funded by the U.S. National Heart, Lung, and Blood Institute that recruited 4,549 participants from Arizona, Oklahoma, and North and South Dakota in 1989–1991 (overall response rate 62%) (Lee et al. 1990). Starting in 1998, an ancillary study to the SHS, the Strong Heart Family Study (SHFS) recruited extended family members of the original SHS participants who were ≥ 18 years of age to evaluate genetic determinants of cardiometabolic disease in American Indian populations (North et al. 2003). For the present study, the population was restricted to SHS participants with measurements of metals in urine at baseline (visit 1) (Scheer et al. 2012) who also participated in the SHFS (North et al. 2003) and had biological samples collected at two follow-up clinic visits conducted in 1993–1995 (visit 2) and 1997–1999 (visit 3). A total of 517 participants met those criteria. To maximize the efficiency of this relatively small epigenetic study (Stuart and Hanna 2013; Zubizarreta et al. 2013), we used a stratified random sample to select 8 participants with moderate arsenic exposure and 8 participants with low arsenic exposure from each region (16 from Arizona, 16 from Oklahoma, and 16 from North or South Dakota), resulting in a total of 48 participants. On the basis of SHS distributions in 1989–1991, we defined relatively low and moderate arsenic exposures of the sum of inorganic and methylated arsenic species as urinary concentrations < 7.2 μg/g (tertile 1) and ≥ 14.0 μg/g (tertile 3), respectively. The protocol for this ancillary study was approved by the Johns Hopkins Bloomberg School of Public Health Institutional Review Board and the Indian Health Service Review Boards and by the participating American Indian communities. All the participants provided oral and written informed consent.

*Epigenetic measurements*. We detected global methylation and global hydroxymethylation in DNA isolated from frozen buffy coat from visit 1 and frozen whole blood from visit 3 using Methylamp (currently known as MethylFlash) Methylated and Hydroxymethylated DNA Quantification Kits (Epigentek) according to the manufacturer’s instructions. In brief, 5-mC and 5-hmC were separately detected using an ELISA-like reaction. Levels of 5-mC or 5-hmC in DNA of all biological samples are reported as the amount of methylated or hydroxymethylated cystosines relative to the cytosine genomic content (percent). For the global DNA methylation assay, the capture antibody for 5-mC had no or negligible cross-reactivity to 5-hmC and unmethylated cytosine. Global DNA hydroxymethylation was quantified by specifically measuring levels of 5-hmC without cross-reactivity to 5-mC and unmethylated cytosine. The input DNA concentrations for the 5-mC and 5-hmC assays were 100 ng and 37.5 ng, respectively. All samples (or repeats) were loaded using the same amount of DNA in the assay plate.

All samples and methylated/hydroxymethylated standards were measured in triplicate, and the average is reported. The observed quality control data were excellent [median (interquartile range; IQR) intra-assay coefficient of variation (CV) ranged from 0.25% (0.10, 0.51) to 0.3% (0.20, 0.69); intraclass correlation coefficient (ICC) > 0.998; see Supplemental Material, Table S1], indicating that the variability of the determinations could be almost completely attributed to between-subjects variation. Leukocyte composition of peripheral blood can covary with patterns of DNA methylation and also with participant characteristics, such as sex or age (Lam et al. 2012). Thus, it was important to account for blood cell heterogeneity in the statistical analysis of DNA methylation and hydroxymethylation data. Blood cell count data, however, was available only for the blood samples corresponding to the DNA extracted in visit 3. Cell counts were available for the following subtypes: neutrophils, lymphocytes, monocytes, eosinophils, and basophils (see distribution in Supplemental Material, Table S2).

*Other variables*. Participants were interviewed and physically examined by centrally trained and certified staff following a standard protocol (Lee et al. 1990). Baseline information on sociodemographic data (age, sex, education), smoking status (never/former/current), cumulative smoking (cigarette pack-years), and alcohol use (never/former/current) were obtained by questionnaire during the interview. Measures of adiposity obtained during the physical exam included body mass index (BMI; kilograms per meter squared), percent body fat estimated by bioelectric impedance (Impedance Meter, model B1A101; RJL Equipment Company), and waist circumference measured supine in centimeters.

Spot urine samples were frozen without chemical additives within 1–2 hr of collection at baseline (visit 1) (Bornhorst et al. 2005). In 2009, urine samples were thawed, and arsenic, cadmium, antimony, and tungsten were measured using inductively coupled plasma-mass spectrometry (ICPMS), as previously described by Scheer et al. (2012). Arsenic species [inorganic arsenic (iAs; arsenite, arsenate), methylarsonate (MMA), and dimethylarsinate (DMA)] were measured in the same urine samples using anion-exchange high-performance liquid chromatography coupled with ICPMS. The limits of detection were 0.1 μg/L for total arsenic and arsenic species, and 0.05 μg/L for cadmium, antimony, and tungsten. None of the samples included in this study were below the limit of detection. The interassay CVs for total arsenic, arsenite, arsenate, MMA, DMA, cadmium, antimony, and tungsten were 4.4, 14.7, 6.9, 6.4, 6.0, 8.7, 30.0, and 14.5%, respectively. For every batch of 79 samples, 10 of the samples were analyzed in duplicate. The mean intra-assay CVs for total arsenic, arsenite, arsenate, MMA, DMA, cadmium, antimony, and tungsten were 1.53, 4.46, 4.23, 3.02, 1.49, 1.34, 3.29, and 0.57%, respectively. The estimated intra-assay ICCs in these samples for total arsenic, arsenite, arsenate, MMA, DMA, cadmium, antimony, and tungsten were 0.997, 0.990, 0.996, 0.992, 0.987, 0.994, 0.966, and 0.990, respectively. To account for urine dilution, urinary metal concentrations (micrograms per liter) were divided by urinary creatinine concentrations (grams per liter) and reported in micrograms per gram creatinine. The Spearman’s correlation coefficients (*r*_s_) for metal-by-metal levels ranged from 0.07 for the correlation between antimony and arsenic to 0.43 for the correlation between tungsten and antimony. To assess arsenic metabolism, we computed the percent iAs (%iAs), %MMA, and %DMA by dividing the concentration of each of them by the sum of the inorganic and methylated species.

*Statistical analyses*. We estimated median (IQR) of %5-mC and %5-hmC levels by participant characteristics. The levels of %5-mC and %5-hmC were not normally distributed and were thus logit-transformed for statistical analyses. Scatter plots, lowess models, and *r*_s_ were used to descriptively display the association between global DNA methylation and global DNA hydroxymethylation at visits 1 and 3. We also examined crude linear trends in the association of global DNA methylation and global DNA hydroxymethylation with continuous variables, including age, BMI, percent body fat, waist circumference, urinary metal concentrations (arsenic, cadmium, tungsten, and antimony), and arsenic metabolic profile (%iAs, %MMA, and %DMA); the corresponding linear correlation coefficients were estimated as the square root of the *R*^2^ of the underlying simple linear regression models. In addition, we used linear regression models on logit-transformed measures of global DNA methylation and global DNA hydroxymethylation to examine associations between methylation and hydroxymethylation levels and categorical variables [sex, education (< 12 years/≥ 12 years), smoking status (ever/never), alcohol status (ever/never), BMI (< 30 kg/m^2^/≥ 30 kg/m^2^)], and continuous variables dichotomized at their corresponding medians (waist circumference, percent body fat, urinary arsenic concentration and metabolism, and urinary concentrations of cadmium, antimony, and tungsten).

Because of limited sample size and difficulties in conducting longitudinal evaluations of changes over time, we performed the analysis separately for visits 1 and 3; results from parsimonious models with no multivariable adjustment were considered the main results. We examined the association of baseline urinary metal concentrations with baseline global DNA methylation and global DNA hydroxymethylation to evaluate the hypothesis that metals are cross-sectionally associated with DNA methylation and hydroxymethylation levels. To evaluate the hypothesis that metals are prospectively related to DNA methylation and hydroxymethylation levels, we examined the association of baseline metal exposure biomarkers with visit 3 global DNA methylation and global DNA hydroxymethylation. For arsenic, under constant conditions of exposure over time, urinary concentrations and metabolism biomarkers have been fairly constant, as previously shown in our study population (Navas-Acien et al. 2009) and in previous studies measuring arsenic in private and public drinking water systems over long periods of time (Karagas et al. 2001; Ryan et al. 2000; Steinmaus et al. 2005). Given this background, evaluating the association of arsenic exposure and metabolism with epigenetic measures in visits 1 and 3 allowed us to evaluate the consistency of the associations assuming constant arsenic exposure and metabolic processes.

In addition to crude regression models (model 1), we also conducted multivariable regression models adjusting for age (years), sex (male, female), BMI (continuous), and smoking status (never, former, and current smokers). To evaluate a potential effect of blood cell type heterogeneity in the associations by participant characteristic, we also adjusted visit 3 models for cell type heterogeneity (models 2 and 3). We had available information on white blood cell count and percent cell type only for visit 3. Because the power in our study is limited and because neutrophils are the cell type more common in blood, we present only results for models adjusted for log-transformed total cell count and percentage of neutrophils. In additional sensitivity analyses, we adjusted for log-transformed total cell count and cell counts of neutrophils, basophils, monocytes, and lymphocytes, with similar findings (data not shown). All statistical analyses were conducted using R software (version R3.1.0; http://www.r-project.org/).

*Post hoc analysis*. The SHS population has high rates of cardiovascular disease. To evaluate 5-mC and 5-hmC levels in a population with a low burden of disease, we detected global methylation and global hydroxymethylation in DNA isolated from frozen whole blood samples from 48 healthy men from Spain (24 were never-smokers and 14 were obese) who participated in the Aragon Workers Health Study (AWHS). The AWHS is a large prospective cohort study that aims to characterize the factors associated with metabolic abnormalities and subclinical atherosclerosis in a middle-aged population free of cardiovascular disease (Casasnovas et al. 2012). The AWHS design and baseline characteristics have been reported elsewhere (Casasnovas et al. 2012). To measure 5-mC and 5-hmC in the AWHS, we used an ELISA method (5-mC and 5-hmC kits; Zymo Research) following the manufacturer’s instructions; we confirmed that the results were consistent in a subsample of the SHS with duplicate determinations (data not shown). Levels of 5-mC or 5-hmC in DNA were measured as the percentage of methylated or hydroxymethylated cystosines in total DNA content (%5-mC and %5-hmC). The input DNA for 5-mC and 5-hmC assays was 50 ng, and all samples (or repeats) were loaded with the same amount of DNA in the assay plate. The intra-assay CVs (IQRs) and ICCs were 3.14% (3.05, 3.20) and 0.86 for %5-mC, and 2.80% (1.40, 4.30) and 0.61 for %5-hmC (see Supplemental Material, Table S1).

## Results

**Figure 1:**
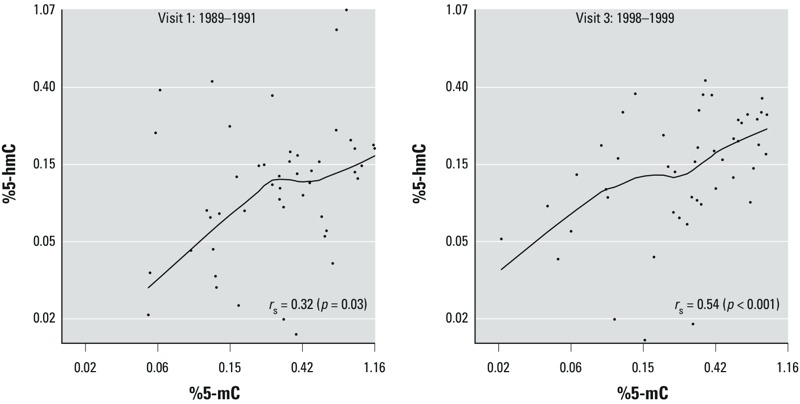
Relationship of global DNA methylation (%5‑mC) and global DNA hydroxymethylation (%5‑hmC) in blood collected in two study visits approximately 10 years apart [Strong Heart Study (SHS)].

The mean (± SD) age of the study sample was 54.9 ± 7.2 years, 68.7% were women, and 58.3% were ever-smokers (Table 1). The study population was representative of the SHS participants who also participated in the SHFS (Table 1). The overall median (IQR) levels of global DNA methylation and global DNA hydroxymethylation were 0.32% (0.15, 0.58) and 0.12% (0.07, 0.17), respectively, for visit 1 and 0.32% (0.13, 0.55) and 0.15% (0.09, 0.25), respectively, for visit 3 (see Supplemental Material, Table S3). For 5-mC and 5-hmC levels, *r*_s_ = 0.32 (*p* = 0.03) at visit 1 and *r*_s_ = 0.54 (*p* < 0.001) at visit 3 (Figure 1).

**Table 1:** Baseline participant characteristics in the Strong Heart Study (SHS).

Characteristic	SHS sample, visit 1 (*n* = 48)	SHS and SHFS^*a*^**(*n* = 517)
Age (years)	54.9 ± 7.2	55.0 ± 7.3
Female	33 (68.7)	343 (66.3)
Education < high school	19 (39.6)	214 (41.4)
BMI (kg/m^2^)^*b*^	31.0 ± 5.8	31.6 ± 6.0
Waist circumference (cm)^*c*^	105.0 ± 14.0	106.7 ±14.3
Percent body fat	36.8 ± 8.8	37.9 ± 8.9
Ever-smokers	28 (58.3)	326 (63.1)
Cumulative smoking (pack-years)^*c*^	8.5 ± 15.5	9.3 ± 17.40
Ever alcohol drinkers	40 (83.3)	421 (81.4)
Urinary arsenic (μg/g)^*d *^	10.02 (6.32, 16.20)	8.10 (5.10, 14.6)
%iAs	8.53 (5.53, 10.71)	7.63 (5.44, 10.30)
%MMA	14.35 (11.02, 17.82)	13.69 (10.60, 16.93)
%DMA	78.32 (71.27, 81.39)	78.69 (72.15, 83.14)
Cadmium (μg/g)	0.88 (0.52, 1.45)	0.92 (0.61, 1.45)
Antimony (μg/g)	0.27 (0.17, 0.46)	0.22 (0.15, 0.36)
Tungsten (μg/g)	0.13 (0.08, 0.27)	0.14 (0.07, 0.25)
NA, not available. Data are mean ± SD, *n* (%), or median (IQR). ^***a***^SHS participants with urinary metal measurements who also participated in the Strong Heart Family Study (SHFS). ^***b***^*n* = 47. ^***c***^*n* = 46. ^***d***^Sum of inorganic and methylated arsenic species in urine.		

The direction of estimated linear trends in 5-mC and 5-hmC levels by levels of possible determinants was generally consistent for both epigenetic modification measures and at both visits (see Supplemental Material, Figures S1 and S2), with some exceptions. In crude cross-sectional analyses, baseline age, %iAs, and %MMA showed a trend toward an inverse association with 5-mC and 5-hmC measured in visit 1 (Table 2; see also Supplemental Material, Figures S1 and S2). Baseline adiposity measures (especially BMI), %DMA, cadmium, antimony, and tungsten showed a trend toward a positive association with 5-mC and 5-hmC measured in visit 1 (Table 2; see also Supplemental Material, Figures S1 and S2). Arsenic metabolism markers (%iAs, %MMA, and %DMA) showed similar direction and magnitude for the cross-sectional and prospective associations with %5-mC and %5-hmC levels (Tables 2 and 3; see also Supplemental Material, Figures S1 and S2). In a comparison of participants with baseline %DMA above and below 78.3%, the odds ratios (ORs) of %5-hmC were 1.75 [95% confidence interval (CI): 1.07, 2.88] at visit 1 and 1.34 (95% CI: 0.79, 2.26) at visit 3 (Table 3). Comparing participants with urinary cadmium concentrations above and below 0.87 μg/g resulted in an OR for %5-mC of 1.64 (95% CI: 1.02, 2.65) at visit 1 but 0.86 (95% CI: 0.47, 1.56) at visit 3 (Table 3).

**Table 2:** Cross-sectional associations [OR (95% CI)] of global DNA methylation (%5‑mC) and global DNA hydroxymethylation (%5‑hmC) in 1989–1991 (visit 1) by participant characteristics in the Strong Heart Study (SHS).

Characteristic	*n*	Methylation		Hydroxymethylation	
		Model 1	Model 2	Model 1	Model 2
Age (years)
< 54	24	1.00 (Ref)	1.00 (Ref)	1.00 (Ref)	1.00 (Ref)
≥ 54	24	0.79 (0.48, 1.30)	0.78 (0.46, 1.32)	0.76 (0.46, 1.26)	0.90 (0.54, 1.50)
Sex
Male	15	1.00 (Ref)	1.00 (Ref)	1.00 (Ref)	1.00 (Ref)
Female	33	1.37 (0.80, 2.33)	1.36 (0.77, 2.38)	1.20 (0.69, 2.07)	1.23 (0.72, 2.10)
Education (years)
≥ 12	29	1.00 (Ref)	1.00 (Ref)	1.00 (Ref)	1.00 (Ref)
< 12	19	1.23 (0.74, 2.05)	1.18 (0.67, 2.08)	0.64 (0.39, 1.07)	0.74 (0.43, 1.26)
BMI (kg/m^2^)
< 30	20	1.00 (Ref)	1.00 (Ref)	1.00 (Ref)	1.00 (Ref)
≥ 30	27	1.35 (0.81, 2.25)	1.35 (0.80, 2.28)	1.20 (0.73, 1.98)	1.17 (0.71, 1.95)
Waist circumference (cm)
< Sex-specific median^*a*^	20	1.00 (Ref)	1.00 (Ref)	1.00 (Ref)	1.00 (Ref)
≥ Sex-specific median	26	0.90 (0.53, 1.53)	0.88 (0.52, 1.50)	1.05 (0.63, 1.75)	0.99 (0.59, 1.65)
Percent body fat
< Sex-specific median^*b*^	23	1.00 (Ref)	1.00 (Ref)	1.00 (Ref)	1.00 (Ref)
≥ Sex-specific median	24	0.94 (0.56, 1.56)	0.90 (0.53, 1.51)	1.47 (0.91, 2.38)	1.58 (0.98, 2.56)
Smoking
Never	20	1.00 (Ref)	1.00 (Ref)	1.00 (Ref)	1.00 (Ref)
Ever	28	0.81 (0.49, 1.35)	0.81 (0.47, 1.38)	1.02 (0.61, 1.72)	1.09 (0.65, 1.82)
Alcohol status
Never	8	1.00 (Ref)	1.00 (Ref)	1.00 (Ref)	1.00 (Ref)
Ever	40	0.97 (0.49, 1.90)	1.06 (0.46, 2.43)	0.85 (0.43, 1.68)	0.71 (0.32, 1.58)
Urinary arsenic (μg/g)^*c *^
< 7.2	24	1.00 (Ref)	1.00 (Ref)	1.00 (Ref)	1.00 (Ref)
≥ 14.0	24	1.05 (0.64, 1.74)	1.05 (0.61, 1.80)	0.74 (0.44, 1.22)	0.73 (0.44, 1.22)
%iAs
< 8.5	23	1.00 (Ref)	1.00 (Ref)	1.00 (Ref)	1.00 (Ref)
≥ 8.5	23	0.85 (0.51, 1.43)	0.78 (0.44, 1.39)	0.56 (0.34, 0.92)	0.61 (0.36, 1.01)
%MMA
< 14.4	23	1.00 (Ref)	1.00 (Ref)	1.00 (Ref)	1.00 (Ref)
≥ 14.4	23	0.73 (0.44, 1.21)	0.78 (0.44, 1.40)	0.59 (0.36, 0.97)	0.71 (0.42, 1.20)
%DMA
< 78.3	23	1.00 (Ref)	1.00 (Ref)	1.00 (Ref)	1.00 (Ref)
≥ 78.3	23	1.56 (0.95, 2.57)	1.64 (0.93, 2.88)	1.76 (1.07, 2.88)	1.59 (0.95, 2.66)
Cadmium (μg/g)
< 0.87	24	1.00 (Ref)	1.00 (Ref)	1.00 (Ref)	1.00 (Ref)
≥ 0.87	24	1.64 (1.02, 2.65)	1.75 (0.96, 3.20)	1.16 (0.70, 1.94)	1.08 (0.59, 1.97)
Antimony (μg/g)
< 0.27	24	1.00 (Ref)	1.00 (Ref)	1.00 (Ref)	1.00 (Ref)
≥ 0.27	24	1.37 (0.84, 2.24)	1.24 (0.71, 2.15)	1.07 (0.64, 1.78)	1.08 (0.64, 1.85)
Tungsten (μg/g)
< 0.13	24	1.00 (Ref)	1.00 (Ref)	1.00 (Ref)	1.00 (Ref)
≥ 0.13	24	1.50 (0.92, 2.45)	1.46 (0.85, 2.52)	1.25 (0.76, 2.08)	1.13 (0.66, 1.92)
%5-mC
< 0.32	24	NA	NA	1.00 (Ref)	1.00 (Ref)
≥ 0.32	24	NA	NA	1.36 (0.82, 2.25)	1.56 (0.95, 2.56)
%5-hmC
< 0.12	23	1.00 (Ref)	1.00 (Ref)	NA	NA
≥ 0.12	25	1.63 (1.01, 2.64)	1.93 (1.15, 3.26)	NA	NA
Abbreviations: NA, not available; Ref, referent. Model 1 was unadjusted; model 2 was adjusted for age (years), sex, smoking status (never, former, current), and BMI (kg/m^2^). ^***a***^Sex-specific medians for waist circumference were 130 cm in men and 130 cm in women. ^***b***^Sex-specific medians for percent body fat were 29.8% in men and 41.40% in women. ^***c***^Sum of inorganic and methylated arsenic species in urine.					

**Table 3:** Prospective associations [OR (95% CI)] of global DNA methylation (%5‑mC) and global DNA hydroxymethylation (%5‑hmC) in 1993–1995 (visit 3) by participant characteristics in the Strong Heart Study (SHS).

Characteristic	*n*	Methylation			Hydroxymethylation		
		Model 1	Model 2	Model 3	Model 1	Model 2	Model 3
Age (years)
< 54	21	1.00 (Ref)	1.00 (Ref)	1.00 (Ref)	1.00 (Ref)	1.00 (Ref)	1.00 (Ref)
≥ 54	23	0.62 (0.34, 1.10)	0.62 (0.35, 1.12)	0.56 (0.31, 1.03)	0.63 (0.38, 1.10)	0.63 (0.38, 1.05)	0.64 (0.37, 1.10)
Sex
Male	15	1.00 (Ref)	1.00 (Ref)	1.00 (Ref)	1.00 (Ref)	1.00 (Ref)	1.00 (Ref)
Female	29	0.76 (0.41, 1.42)	0.80 (0.42, 1.53)	0.69 (0.36, 1.33)	1.20 (0.70, 2.08)	1.24 (0.70, 2.18)	1.16 (0.66, 2.04)
Education (years)
≥ 12	27	1.00 (Ref)	1.00 (Ref)	1.00 (Ref)	1.00 (Ref)	1.00 (Ref)	1.00 (Ref)
< 12	17	1.08 (0.58, 1.99)	1.06 (0.56, 2.01)	1.07 (0.54, 2.09)	1.20 (0.71, 2.05)	1.17 (0.67, 2.04)	1.23 (0.69, 2.19)
BMI (kg/m^2^)
< 30	18	1.00 (Ref)	1.00 (Ref)	1.00 (Ref)	1.00 (Ref)	1.00 (Ref)	1.00 (Ref)
≥ 30	25	1.67 (0.92, 3.05)	1.69 (0.91, 3.14)	1.69 (0.92, 3.10)	1.05 (0.61, 1.80)	1.08 (0.62, 1.89)	1.01 (0.59, 1.73)
Waist circumference (cm)
< Sex-specific median^*a*^	19	1.00 (Ref)	1.00 (Ref)	1.00 (Ref)	1.00 (Ref)	1.00 (Ref)	1.00 (Ref)
≥ Sex-specific median	24	1.19 (0.64, 2.20)	1.16 (0.63, 2.17)	1.16 (0.63, 2.14)	0.86 (0.51, 1.47)	0.85 (0.50, 1.47)	0.82 (0.49, 1.38)
Percent body fat
< Sex-specific median^*b*^	21	1.00 (Ref)	1.00 (Ref)	1.00 (Ref)	1.00 (Ref)	1.00 (Ref)	1.00 (Ref)
≥ Sex-specific median	22	1.61 (0.89, 2.92)	1.60 (0.87, 2.93)	1.45 (0.79, 2.65)	1.48 (0.88, 2.49)	1.50 (0.89, 2.55)	1.41 (0.84, 2.36)
Smoking
Never	19	1.00 (Ref)	1.00 (Ref)	1.00 (Ref)	1.00 (Ref)	1.00 (Ref)	1.00 (Ref)
Ever	25	0.58 (0.32, 1.03)	0.61 (0.33, 1.13)	0.51 (0.27, 0.98)	0.69 (0.41, 1.16)	0.71 (0.41, 1.24)	0.68 (0.39, 1.18)
Alcohol status
Never	8	1.00 (Ref)	1.00 (Ref)	1.00 (Ref)	1.00 (Ref)	1.00 (Ref)	1.00 (Ref)
Ever	36	0.86 (0.40, 1.87)	0.84 (0.38, 1.85)	0.93 (0.35, 2.44)	0.89 (0.45, 1.75)	0.89 (0.45, 1.78)	0.90 (0.39, 2.07)
Urinary arsenic (μg/g)^*c *^
< 7.2	23	1.00 (Ref)	1.00 (Ref)	1.00 (Ref)	1.00 (Ref)	1.00 (Ref)	1.00 (Ref)
≥ 14.0	21	0.58 (0.33, 1.03)	0.54 (0.30, 0.97)	0.59 (0.32, 1.10)	0.78 (0.46, 1.30)	0.73 (0.43, 1.25)	0.80 (0.46, 1.39)
%iAs
< 8.5	21	1.00 (Ref)	1.00 (Ref)	1.00 (Ref)	1.00 (Ref)	1.00 (Ref)	1.00 (Ref)
≥ 8.5	22	0.91 (0.50, 1.68)	0.90 (0.48, 1.67)	0.78 (0.40, 1.50)	0.83 (0.49, 1.40)	0.79 (0.46, 1.37)	0.71 (0.40, 1.25)
%MMA
< 14.4	21	1.00 (Ref)	1.00 (Ref)	1.00 (Ref)	1.00 (Ref)	1.00 (Ref)	1.00 (Ref)
≥ 14.4	22	0.73 (0.40, 1.34)	0.73 (0.40, 1.34)	0.70 (0.36, 1.37)	0.58 (0.35, 0.96)	0.58 (0.35, 0.98)	0.62 (0.35, 1.10)
%DMA
< 78.3	22	1.00 (Ref)	1.00 (Ref)	1.00 (Ref)	1.00 (Ref)	1.00 (Ref)	1.00 (Ref)
≥ 78.3	21	1.57 (0.87, 2.85)	1.61 (0.88, 2.94)	1.75 (0.92, 3.32)	1.34 (0.79, 2.26)	1.38 (0.81, 2.36)	1.32 (0.75, 2.35)
Cadmium (μg/g)
< 0.87	24	1.00 (Ref)	1.00 (Ref)	1.00 (Ref)	1.00 (Ref)	1.00 (Ref)	1.00 (Ref)
≥ 0.87	20	0.86 (0.47, 1.56)	0.90 (0.49, 1.66)	1.03 (0.50, 2.11)	1.04 (0.62, 1.76)	1.07 (0.62, 1.83)	0.97 (0.52, 1.80)
Antimony (μg/g)
< 0.27	22	1.00 (Ref)	1.00 (Ref)	1.00 (Ref)	1.00 (Ref)	1.00 (Ref)	1.00 (Ref)
≥ 0.27	22	1.71 (0.96, 3.04)	1.93 (1.07, 3.47)	2.15 (1.15, 4.01)	1.15 (0.69, 1.94)	1.22 (0.71, 2.10)	1.16 (0.65, 2.07)
Tungsten (μg/g)
< 0.13	23	1.00 (Ref)	1.00 (Ref)	1.00 (Ref)	1.00 (Ref)	1.00 (Ref)	1.00 (Ref)
≥ 0.13	21	0.84 (0.46, 1.52)	0.90 (0.47, 1.71)	0.93 (0.46, 1.86)	1.25 (0.75, 2.10)	1.40 (0.81, 2.44)	1.32 (0.73, 2.37)
%5-mC
< 0.32	22	NA	NA	NA	1.00 (Ref)	1.00 (Ref)	1.00 (Ref)
≥ 0.32	22	NA	NA	NA	1.24 (0.74, 2.08)	1.21 (0.71, 2.06)	1.15 (0.67, 1.96)
%5-hmC
< 0.12	20	1.00 (Ref)	1.00 (Ref)	1.00 (Ref)	NA	NA	NA
≥ 0.12	24	1.23 (0.68, 2.24)	1.16 (0.63, 2.16)	1.28 (0.67, 2.45)	NA	NA	NA
Abbreviations: NA, not available; Ref, referent. Model 1 was unadjusted; model 2 was adjusted for log-transformed total count of white blood cells and percent of neutrophils; and model 3 was adjusted as for model 2 and also adjusted for age (years), sex, smoking status (never, former, current), and BMI (kg/m^2^). ^***a***^Sex-specific medians for waist circumference were 130 cm in men and 130 cm in women. ^***b***^Sex-specific medians for percent body fat were 29.8% in men and 41.40% in women. ^***c***^Sum of inorganic and methylated arsenic species in urine.							

In general, adjustment for age, sex, BMI, and smoking status did not change the direction of the observed associations. Actually, the correlation of %5-mC and %5-hmC became stronger after those adjustments (Table 2). In sensitivity analyses adjusting for cell heterogeneity in the subset of individuals with cell count data available for visit 3 (*n* = 44) (Table 3), results were consistent, although some additional prospective trends became statistically significant. The ORs of %5-mC comparing participants with urinary arsenic > 14.0 μg/g and < 7.2 μg/g, and with urinary antimony above and below 0.27 μg/g, were 0.54 (95% CI: 0.30, 0.97) and 1.93 (95% CI: 1.07, 3.47), respectively. The ORs of %5-hmC comparing participants with baseline %MMA above and below 14.4% was 0.58 (95% CI: 0.35, 0.98).

In a post hoc analysis of the AWHS, the median %5-mC and %5-hmC levels were 0.90% and 0.09%, respectively. For %5-mC and %5-mC, the correlation *r*_s_ = 0.16 (*p* = 0.29) (see Supplemental Material, Figure S3).

## Discussion

Global DNA methylation and global DNA hydroxymethylation measured in blood were moderately and positively associated in this subsample of participants from the SHS. We found consistent associations in our study population at two time points approximately 10 years apart, and also in an independent study population from Spain with a low burden of disease (AWHS); these associations support the close relationship between both epigenetic measures. Although our sample size was limited, we found statistically significant associations between urinary cadmium concentrations and global DNA methylation and between arsenic metabolism (measured as %DMA) and global DNA hydroxymethylation. The observed associations between arsenic metabolism and global DNA methylation and DNA hydroxymethylation showed consistent patterns across time. This study provided the opportunity to evaluate the consistency of potential associations and direction of the relationship between these two measures of epigenetic modification in human blood DNA samples.

Methylation at the 5´ position of cytosine in DNA plays a role in regulating gene expression (Feinberg 2010). In addition to DNA methylation, DNA hydroxymethylation has also been related to changes in gene expression (Branco et al. 2012). In the mammalian genome, Ten to Eleven Translocation (TET) proteins are responsible for catalyzing 5-mC oxidation to 5-hmC (Branco et al. 2012; Tahiliani et al. 2009). Hydroxymethylation at 5´ CpGs via TETs has been shown to contribute to gene transcription by influencing DNA demethylation (Baubec et al. 2013; Shen et al. 2013; Song et al. 2013) and/or recruitment of transcription complexes to repress gene transcription (Cimmino et al. 2011). Bisulfite conversion, a method traditionally used for the enrichment of 5-mC, cannot distinguish between 5-mC and 5-hmC (Huang et al. 2010). Before the development of 5-hmC profiling strategies, it was postulated that hypermethylation of promoter regions blocks gene expression, whereas hypermethylation of gene bodies increases gene expression (Jones 2012). Recent studies evaluating genome-wide 5-hmC profiles in mouse and human embryonic stem cells and brain cells have observed an enrichment of 5-hmC at gene body regions, regulatory (promoter) regions, and sites with intermediate CpG density (Branco et al. 2012). The presence of 5-hmC in gene bodies has been consistently associated with gene expression (Branco et al. 2012). Altogether, the accumulated evidence suggests that gene-specific hydroxymethylation has dual functions in the regulation of gene transcription (Branco et al. 2012). Ficz et al. (2011) proposed that the balance between DNA methylation and DNA hydroxymethylation in the genome is involved in the balance between cellular pluripotency and lineage commitment. The health implications of the relationship between DNA methylation and hydroxymethylation in differentiated tissues, however, are currently unknown. Advanced technology that uses massive parallel sequencing on profiling degree of 5-hmC across the genome may help to understand the role of DNA hydroxymethylation.

In our study population, the levels of 5-hmC were approximately 2.5-fold lower than those of 5-mC (see Supplemental Material, Table S3). In a study by Figueroa-Romero et al. (2012), the mean %5-mC and %5-hmC in blood samples from 12 healthy individuals from the United States, as measured by an ELISA method, were 0.41% and 0.03%, respectively (5-hmC was ~ 12-fold lower than 5-mC). The CV and ICC of the assays were not provided. In the AWHS, the ratio of 5-mC and 5-hmC levels was intermediate compared with the SHS results and with data from Figueroa-Romero et al. (2012); in the AWHS, we found that the level of 5-mC was about 10 times that of 5-hmC (see “Results”). In the SHS data, with very low CVs and high ICCs (see Supplemental Material, Table S1), the correlation between 5-mC and 5-hmC was statistically significant at two time points (Figure 1). In the AWHS, the replication study sample, the correlation between global DNA methylation and global DNA hydroxymethylation was positive, supporting consistency in the direction of the association in a human population with a different risk profile. The correlation, however, was weaker and not statistically significant. Overall, random sampling variability due to the small sample size and technical variability/measurement error cannot be discarded as the main reason for the discrepancies in global DNA methylation and hydroxymethylation levels from human blood DNA samples in different study populations.

Changes in DNA methylation have been related to environmental exposures such as metals, air pollution, benzene, bisphenol A, diethylstilbestrol, and dioxins in experimental and small population-based studies, although the exact mechanisms remain unclear (Bailey and Fry 2014; Breton and Marutani 2014; Hou et al. 2012). In the present study, we found consistent trends for both global DNA methylation and global hydroxymethylation by different determinants. It is possible that DNA hydroxymethylation acts as a proxy for DNA methylation or vice versa (the more DNA methylation, the greater potential for DNA hydroxymethylation). Indeed, Ficz et al. (2011) reported that 5-hmC was reduced in *Tet1/2* knockdown cells and *Np95^–/–^* cells, and eliminated in *Dnmt1^–/–^*/*Dnmt3a^–/–^*/*Dnmt3b^–/–^* triple knockout embryonic stem cells, suggesting that most 5-hmC in the genome depends on preexisting 5-mC. Alternatively, it is also possible that determinants for DNA methylation and DNA hydroxymethylation are somewhat common. For instance, based on *in vivo* and *in vitro* experimental findings, it has been hypothesized that oxidative stress can regulate both DNA methylation and DNA hydroxymethylation processes by impairing one-carbon (Lee et al. 2009) and citric acid metabolism (Chia et al. 2011) pathways, respectively. Additional studies with larger sample sizes are needed to investigate the correlation between global and gene-specific DNA methylation and DNA hydroxymethylation in relation to environmental determinants and health outcomes.

In our study, we found some support for the relationship between some environmental exposures and global DNA methylation and global DNA hydroxymethylation. In particular, we found a change in the level of global DNA methylation and hydroxymethylation associated with metals exposure. For antimony and tungsten, we found positive cross-sectional associations with global DNA methylation and hydroxymethylation, although the association with global DNA hydroxymethylation was weaker. The prospective association of antimony and global DNA methylation was statistically significant. The association of global DNA methylation and hydroxymethylation with these metals has not previously been evaluated in human studies. Very few studies have evaluated the association of arsenic and cadmium exposure with global DNA methylation in humans. Contrary to our findings, Hossain et al. (2012) found that low-level environmental cadmium exposure was associated with global DNA hypomethylation, as measured in repetitive elements (a proxy for global DNA methylation), in women from Argentina (*n* = 202). In a population from Spain (*n* = 892), increasing arsenic toenail concentrations were significantly associated with decreasing methylation of LINE-1 (long interspersed nucleotide element-1) (Tajuddin et al. 2013); toenail cadmium was not associated with LINE-1 methylation in this study population. In populations exposed to high arsenic levels in drinking water in West Bengal and Bangladesh, increasing arsenic exposure levels were associated with increasing global DNA methylation in peripheral blood cells (Majumdar et al. 2010; Pilsner et al. 2007). The association of maternal urinary arsenic and global methylation as measured in Alu and LINE-1 repetitive elements and in the LUMA assay in cord blood DNA was positive among male newborns (*n* = 58) but inverse among female newborns (*n* = 43) from Bangladesh (Pilsner et al. 2012). At low to moderate levels of arsenic exposure, increasing arsenic levels in toenails have been associated with methylation changes in repetitive elements (increasing *Alu* and decreasing *LINE-1* DNA methylation) in 581 elderly men from the United States (Lambrou et al. 2012). In visit 3 data, after adjustment for cell heterogeneity, higher arsenic exposure levels were significantly associated with decreased global DNA methylation. Random sampling variability and differences in residual confounding, study designs, and population exposure levels may underlie inconsistencies across studies evaluating metal-related global methylation.

Arsenic exposure has been associated with both hypermethylation and hypomethylation of gene-specific promoter, with a trend toward hypermethylation (Gribble et al. 2014; Hossain et al. 2012; Kile et al. 2012; Koestler et al. 2013). In 202 Argentinean women, urinary arsenic concentrations were positively associated with methylation of *p16* and *MLH1* genes (Hossain et al. 2012). CpG sites in gene *p16* were also positively associated with arsenic exposure in 113 women from Bangladesh (Kile et al. 2012). In a genome-wide study of DNA methylation in cord blood samples from 134 infants, 75% of the 44 top statistically significant arsenic-associated CpG islands showed increased methylation with increased arsenic exposure levels (Koestler et al. 2013). A hypomethylated region in the *AS3MT* promoter was associated with higher arsenic exposure in our study population (Gribble et al. 2014). Large-sample-size epidemiologic studies are needed to evaluate the relationship of metal exposures with global DNA and gene-specific methylation and hydroxymethylation in human populations.

Arsenic metabolism in humans is usually studied as the relative amount of inorganic and methylated arsenic metabolites in urine (Vahter 2000). Differences in arsenic methylation patterns in urine (higher %iAs and %MMA and lower %DMA) have been associated with a higher risk of skin lesions, cancer, and cardiovascular disease in populations exposed to arsenic in drinking water (Chen et al. 2003; Del Razo et al. 1997; Hsueh et al. 1997; Kile et al. 2011; Steinmaus et al. 2006; Wu et al. 2006; Yu et al. 2000). In a small study population from Mexico (*n* = 16), arsenic species were associated with gene-specific promoter methylation in 812 genes (Bailey et al. 2013). In that study, increasing absolute levels of iAs and MMA were mostly associated with decreasing levels of promoters’ DNA methylation, whereas increasing levels of DMA were inconsistently associated with both increasing and decreasing methylation of specific promoters (Bailey et al. 2013). The interpretation of these findings is unclear because absolute levels of arsenic metabolites depend not only on arsenic metabolism capability but also on arsenic exposure levels. The association of arsenic metabolism and global DNA methylation—measured as %iAs, %MMA, and %DMA—has seldom been explored. In the present study, the relationships of global DNA methylation and global DNA hydroxymethylation with %iAs, %MMA, and %DMA were consistent in the cross-sectional and prospective analyses. Individuals with higher %DMA (faster methylators of inorganic arsenic) also had higher global DNA methylation and global DNA hydroxymethylation, whereas those with higher %iAs and %MMA (slower methylators) tended to have lower global DNA methylation and global DNA hydroxymethylation.

Both DNA methylation and arsenic metabolism require *S*-adenosylmethionine (SAM) as the methyl donor. Competitive demand between arsenic metabolism and DNA methylation for SAM could affect DNA methylation status throughout the genome (Lee et al. 2009). It is also possible that the arsenic methylation profile reflects general methylation capability in the body or that common enzymes are involved in both arsenic and DNA methylation processes. In a recent genome-wide linkage scan on arsenic metabolism in our study population (Tellez-Plaza et al. 2013), we found suggestive peaks near genes encoding several methyltransferases, including the genes *PRDM9* (PR domain zinc finger protein 9, a protein with histone methyltransferase activity) and *EHMT1* (histone methyltransferase). Other minor, borderline-suggestive peaks were near the genes *AS3MT* (arsenic (III) methyltransferase), *METTL20* (methyltransferase-like 20), and *RNMT* (RNA methyltransferase). In a study of 103 Argentinean women and 127 women from Bangladesh delivering singleton infants, *AS3MT* haplotypes associated with efficient arsenic metabolism were also associated with DNA methylation and gene expression of *AS3MT* and other genes on chromosome 10 (Engstrom et al. 2013). In that study, which assayed DNA methylation on a genome-wide basis by using a microarray technology, the association of *AS3MT* efficiency and arsenic metabolism and DNA methylation status in other chromosomes and genomic regions was not reported.

Evidence for plausible mechanisms for effects of metals other than the connection between arsenic and DNA-methylation metabolism on DNA methylation is relatively scarce. Experimental *ex vivo* evidence using M.SssI DNMT (a bacterial DNMT that recognizes the same sequence as mammalian DNMTs) showed that cadmium exposure was an effective, noncompetitive inhibitor of DNMT (Takiguchi et al. 2003). In rat liver cells, short-term cadmium exposure induced DNA global hypomethylation; however, prolonged exposure resulted in global DNA hypermethylation (Takiguchi et al. 2003). Results of other studies are consistent with these findings (Benbrahim-Tallaa et al. 2007; Jiang et al. 2008; Poirier and Vlasova 2002). Metals could indirectly impair both one-carbon (Lee et al. 2009) and citric acid metabolic (Chia et al. 2011) pathways. There is evidence suggesting that TETs are regulated by redox reaction (Dao et al. 2014). TET proteins were activated in the presence of high oxygen levels and alpha-ketoglutarate (α-KG), which is generated in citric acid cycle (Chowdhury et al. 2011; Xu et al. 2011). It has been suggested that imbalance of redox reaction in cells in response to environmental stress or toxicants may affect the ratio of oxidants and eventually alter production of α-KG and activation of TETs (Chia et al. 2011; Dao et al. 2014). Whether metal exposure–induced oxidative stress (Prozialeck et al. 2008; Valko et al. 2005) affects TET-mediated hydroxymethylation is still unknown. It is also possible that, in addition to cadmium, other divalent metals can inhibit DNMT and other enzymes involved not only in one-carbon metabolism and citric acid metabolism pathways but also in histone acetylation, deacetylation, and methylation pathways (Chervona and Costa 2012; Dai and Wang 2014). More mechanistic research is needed for evaluating the role of metals in inducing DNA-methylation changes.

The major limitation of the present study is the small sample size. Another limitation is the tissue-dependent nature of DNA methylation and DNA hydroxymethylation changes. We measured global methylation and global hydroxymethylation in blood cell DNA, which is composed of different cell types, each with a different DNA methylation profile. Information on blood cell counts was available only for visit 3. Thus, for visit 3 we could incorporate information on cell heterogeneity in the analysis, which showed largely consistent and somewhat stronger associations (Table 3, model 2). DNA methylation markers measured in blood samples, moreover, have been related to immunologic disease, mental disease, cardiovascular disease, and cancer, suggesting that blood cells can be an adequate tissue for conducting epigenetic studies in epidemiologic samples (Terry et al. 2011). Finally, creatinine may be a surrogate for several key mediators of the DNA methylation and arsenic metabolism processes because DNA methylation, arsenic methylation (Lee et al. 2009; Loenen 2006), and creatine synthesis (Brosnan et al. 2011) use SAM as the methyl donor. Creatinine, a break-down product of creatine phosphate in muscle, is generally produced at a constant rate depending on muscle mass (Heymsfield et al. 1983). Experimental models are needed to evaluate whether the connection between DNA methylation and arsenic metabolism and measures of body composition are related to muscle mass.

The strengths of the present study include the availability of information in several potential determinants of global DNA methylation and hydroxymethylation, including metals and arsenic metabolism and the high quality of standardized protocols for the recruitment of participants, conduction of interviews, physical examinations, collection of biological samples, and laboratory analysis. Importantly, American Indian populations are an important ethnic group that has often been understudied. Findings from American Indian populations have proven to be applicable to other groups with high rates of diabetes mellitus and obesity (Fretts AM et al. 2012; Howard et al. 1999; Xu et al. 2012). Our findings can thus be relevant to many populations in the United States and around the world who are increasingly affected by the obesity and diabetes epidemics. Finally, in addition to evaluating determinants of traditional global DNA methylation, we also evaluated determinants of global DNA hydroxymethylation, which is a novel and relatively unknown epigenetic marker.

## Conclusions

We found a positive correlation between global DNA methylation and global DNA hydroxymethylation in human blood samples collected in the same individuals at two time points, with confirmation of findings in an independent population with a low burden of disease, supporting that both epigenetic measures are related at the population level. The consistency in the trend of the associations between these epigenetic modifications and categories of available determinants, especially arsenic exposure and metabolism, suggests the need for understanding which of the two measures is a better biomarker for environmental epigenetic effects in future large-scale epidemiologic studies.

## Supplemental Material

(1.3 MB) PDF

## References

[r1] Andrew AS Jewell DA Mason RA Whitfield ML Moore JH Karagas MR 2008 Drinking-water arsenic exposure modulates gene expression in human lymphocytes from a U.S. population. Environ Health Perspect 116 524 531; 10.1289/ehp.10861 18414638 PMC2290973

[r2] Arita A, Costa M (2009). Epigenetics in metal carcinogenesis: nickel, arsenic, chromium and cadmium.. Metallomics.

[r3] Bailey KA, Fry RC (2014). Arsenic-associated changes to the epigenome: what are the functional consequences?. Curr Environ Health Rep.

[r4] Bailey KA, Wu MC, Ward WO, Smeester L, Rager JE, Garcia-Vargas G (2013). Arsenic and the epigenome: interindividual differences in arsenic metabolism related to distinct patterns of DNA methylation.. J Biochem Mol Toxicol.

[r5] Baubec T, Ivanek R, Lienert F, Schubeler D (2013). Methylation-dependent and -independent genomic targeting principles of the MBD protein family.. Cell.

[r6] Benbrahim-Tallaa L Waterland RA Dill AL Webber MM Waalkes MP 2007 Tumor suppressor gene inactivation during cadmium-induced malignant transformation of human prostate cells correlates with overexpression of *de novo* DNA methyltransferase. Environ Health Perspect 115 1454 1459; 10.1289/ehp.10207 17938735 PMC2022656

[r7] Bornhorst JA, Hunt JW, Urry FM, McMillin GA (2005). Comparison of sample preservation methods for clinical trace element analysis by inductively coupled plasma mass spectrometry.. Am J Clin Pathol.

[r8] Bourdonnay E, Morzadec C, Sparfel L, Galibert MD, Jouneau S, Martin-Chouly C (2009). Global effects of inorganic arsenic on gene expression profile in human macrophages.. Mol Immunol.

[r9] Branco MR, Ficz G, Reik W (2012). Uncovering the role of 5-hydroxymethylcytosine in the epigenome.. Nat Rev Genet.

[r10] Breton CV, Marutani AN (2014). Air pollution and epigenetics: recent findings.. Curr Environ Health Rep.

[r11] Brosnan JT, da Silva RP, Brosnan ME (2011). The metabolic burden of creatine synthesis.. Amino Acids.

[r12] Casasnovas JA Alcaide V Civeira F Guallar E Ibañez B Borreguero JJ 2012 Aragon Workers’ Health Study—design and cohort description. BMC Cardiovasc Disord 12 45; 10.1186/1471-2261-12-45 22712826 PMC3439398

[r13] Castillo P Ibañez F Guajardo A Llanos MN Ronco AM 2012 Impact of cadmium exposure during pregnancy on hepatic glucocorticoid receptor methylation and expression in rat fetus. PloS One 7 e44139; 10.1371/journal.pone.0044139 22957049 PMC3434215

[r14] Chen YC, Su HJ, Guo YL, Hsueh YM, Smith TJ, Ryan LM (2003). Arsenic methylation and bladder cancer risk in Taiwan.. Cancer Causes Control.

[r15] Cheng TF, Choudhuri S, Muldoon-Jacobs K (2012). Epigenetic targets of some toxicologically relevant metals: a review of the literature.. J Appl Toxicol.

[r16] Chervona Y, Costa M (2012). The control of histone methylation and gene expression by oxidative stress, hypoxia, and metals.. Free Radic Biol Med.

[r17] Chia N, Wang L, Lu X, Senut MC, Brenner C, Ruden DM (2011). Hypothesis: environmental regulation of 5-hydroxymethylcytosine by oxidative stress.. Epigenetics.

[r18] Chowdhury R, Yeoh KK, Tian YM, Hillringhaus L, Bagg EA, Rose NR (2011). The oncometabolite 2-hydroxyglutarate inhibits histone lysine demethylases.. EMBO Rep.

[r19] Cimmino L, Abdel-Wahab O, Levine RL, Aifantis I (2011). TET family proteins and their role in stem cell differentiation and transformation.. Cell Stem Cell.

[r20] Dai H, Wang Z (2014). Histone modification patterns and their responses to environment.. Curr Environ Health Rep.

[r21] Dao T, Cheng RYS, Revelo MP, Mitzner W, Tang WT (2014). Hydroxymethylation as a novel environmental biosensor.. Curr Environ Health Rep.

[r22] Del Razo LM, Garcia-Vargas GG, Vargas H, Albores A, Gonsebatt ME, Montero R (1997). Altered profile of urinary arsenic metabolites in adults with chronic arsenicism. A pilot study.. Arch Toxicol.

[r23] Engstrom KS Hossain MB Lauss M Ahmed S Raqib R Vahter M 2013 Efficient arsenic metabolism—the *AS3MT* haplotype is associated with DNA methylation and expression of multiple genes around *AS3MT*. PloS One 8 e53732; 10.1371/journal.pone.005373 23341986 PMC3544896

[r24] Feinberg AP (2010). Genome-scale approaches to the epigenetics of common human disease.. Virchows Archiv.

[r25] Ficz G Branco MR Seisenberger S Santos F Krueger F Hore TA 2011 Dynamic regulation of 5-hydroxymethylcytosine in mouse ES cells and during differentiation [Letter] Nature 473 398 402 21460836 10.1038/nature10008

[r26] Figueroa-Romero C Hur J Bender DE Delaney CE Cataldo MD Smith AL 2012 Identification of epigenetically altered genes in sporadic amyotrophic lateral sclerosis. PloS One 7 e52672; 10.1371/journal.pone.0052672 23300739 PMC3530456

[r27] Fretts AM, Howard BV, McKnight B, Duncan GE, Beresford SA, Calhoun D (2012). Modest levels of physical activity are associated with a lower incidence of diabetes in a population with a high rate of obesity: the Strong Heart Family Study.. Diabetes Care.

[r28] Gribble MO, Tang W, Shang T, Pollak J, Umans JG, Francesconi KA (2014). Differential methylation of the arsenic (III) methyltransferase promoter according to arsenic exposure.. Arch Toxicol.

[r29] Heymsfield SB, Arteaga C, McManus C, Smith J, Moffitt S (1983). Measurement of muscle mass in humans: validity of the 24-hour urinary creatinine method.. Am J Clin Nutr.

[r30] Hossain MB Vahter M Concha G Broberg K 2012 Low-level environmental cadmium exposure is associated with DNA hypomethylation in Argentinean women. Environ Health Perspect 120 879 884; 10.1289/ehp.1104600 22382075 PMC3385444

[r31] Hou L, Zhang X, Wang D, Baccarelli A (2012). Environmental chemical exposures and human epigenetics.. Int J Epidemiol.

[r32] Howard BV, Lee ET, Cowan LD, Devereux RB, Galloway JM, Go OT (1999). Rising tide of cardiovascular disease in American Indians. The Strong Heart Study.. Circulation.

[r33] Hsueh YM, Chiou HY, Huang YL, Wu WL, Huang CC, Yang MH (1997). Serum β-carotene level, arsenic methylation capability, and incidence of skin cancer.. Cancer Epidemiol Biomarkers Prev.

[r34] Huang D, Zhang Y, Qi Y, Chen C, Ji W (2008). Global DNA hypomethylation, rather than reactive oxygen species (ROS), a potential facilitator of cadmium-stimulated K562 cell proliferation.. Toxicol Lett.

[r35] Huang Y Pastor WA Shen Y Tahiliani M Liu DR Rao A 2010 The behaviour of 5-hydroxymethylcytosine in bisulfite sequencing. PloS one 5 e8888; 10.1371/journal.pone.0008888 20126651 PMC2811190

[r36] Jiang G, Xu L, Song S, Zhu C, Wu Q, Zhang L (2008). Effects of long-term low-dose cadmium exposure on genomic DNA methylation in human embryo lung fibroblast cells.. Toxicology.

[r37] Jones PA (2012). Functions of DNA methylation: islands, start sites, gene bodies and beyond.. Nat Rev Genet.

[r38] Karagas MR, Le CX, Morris S, Blum J, Lu X, Spate V (2001). Markers of low level arsenic exposure for evaluating human cancer risks in a US population.. Int J Occup Men Environ Health.

[r39] Kile ML Baccarelli A Hoffman E Tarantini L Quamruzzaman Q Rahman M 2012 Prenatal arsenic exposure and DNA methylation in maternal and umbilical cord blood leukocytes. Environ Health Perspect 120 1061 1066; 10.1289/ehp.1104173 22466225 PMC3404653

[r40] Kile ML, Hoffman E, Rodrigues EG, Breton CV, Quamruzzaman Q, Rahman M (2011). A pathway-based analysis of urinary arsenic metabolites and skin lesions.. Am J Epidemiol.

[r41] Koestler DC Avissar-Whiting M Houseman EA Karagas MR Marsit CJ 2013 Differential DNA methylation in umbilical cord blood of infants exposed to low levels of arsenic *in utero*. Environ Health Perspect 121 971 977; 10.1289/ehp.1205925 23757598 PMC3733676

[r42] Lam LL, Emberly E, Fraser HB, Neumann SM, Chen E, Miller GE (2012). Factors underlying variable DNA methylation in a human community cohort.. Proc Nat Acad Sci USA.

[r43] Lambrou A, Baccarelli A, Wright RO, Weisskopf M, Bollati V, Amarasiriwardena C (2012). Arsenic exposure and DNA methylation among elderly men.. Epidemiology.

[r44] Lee DH Jacobs DR Jr Porta M 2009 Hypothesis: a unifying mechanism for nutrition and chemicals as lifelong modulators of DNA hypomethylation. Environ Health Perspect 117 1799 1802; 10.1289/ehp.0900741 20049195 PMC2799450

[r45] Lee ET, Welty TK, Fabsitz R, Cowan LD, Le NA, Oopik AJ (1990). The Strong Heart Study. A study of cardiovascular disease in American Indians: design and methods.. Am J Epidemiol.

[r46] Loenen WA (2006). *S*-Adenosylmethionine: jack of all trades and master of everything?. Biochem Soc Trans.

[r47] Majumdar S, Chanda S, Ganguli B, Mazumder DN, Lahiri S, Dasgupta UB (2010). Arsenic exposure induces genomic hypermethylation.. Environ Toxicol.

[r48] Navas-Acien A Umans JG Howard BV Goessler W Francesconi KA Crainiceanu CM 2009 Urine arsenic concentrations and species excretion patterns in American Indian communities over a 10-year period: the Strong Heart Study. Environ Health Perspect 117 1428 1433; 10.1289/ehp.0800509 19750109 PMC2737021

[r49] North KE, Howard BV, Welty TK, Best LG, Lee ET, Yeh JL (2003). Genetic and environmental contributions to cardiovascular disease risk in American Indians: the Strong Heart Family study.. Am J Epidemiol.

[r50] Ordovas JM, Smith CE (2010). Epigenetics and cardiovascular disease.. Nat Rev Cardiol.

[r51] Pilsner JR Hall MN Liu X Ilievski V Slavkovich V Levy D 2012 Influence of prenatal arsenic exposure and newborn sex on global methylation of cord blood DNA. PloS One 7 e37147; 10.1371/journal.pone.0037147 22662134 PMC3360698

[r52] Pilsner JR, Liu X, Ahsan H, Ilievski V, Slavkovich V, Levy D (2007). Genomic methylation of peripheral blood leukocyte DNA: influences of arsenic and folate in Bangladeshi adults.. Am J Clin Nutr.

[r53] Poirier LA, Vlasova TI (2002). The prospective role of abnormal methyl metabolism in cadmium toxicity.. Environ Health Perspect.

[r54] Prozialeck WC, Edwards JR, Nebert DW, Woods JM, Barchowsky A, Atchison WD (2008). The vascular system as a target of metal toxicity.. Toxicol Sci.

[r55] Reichard JF, Puga A (2010). Effects of arsenic exposure on DNA methylation and epigenetic gene regulation.. Epigenomics.

[r56] Ren X McHale CM Skibola CF Smith AH Smitm MT Zhang L 2011 An emerging role for epigenetic dysregulation in arsenic toxicity and carcinogenesis. Environ Health Perspect 119 11 19; 10.1289/ehp.1002114 20682481 PMC3018488

[r57] Ryan PB, Huet N, MacIntosh DL (2000). Longitudinal investigation of exposure to arsenic, cadmium, and lead in drinking water.. Environ Health Perspect.

[r58] Scheer J, Findenig S, Goessler W, Francesconi KA, Howard B, Umans JG (2012). Arsenic species and selected metals in human urine: validation of HPLC/ICPMS and ICPMS procedures for a long-term population-based epidemiological study.. Anal Methods.

[r59] Shen L, Wu H, Diep D, Yamaguchi S, D’Alessio AC, Fung HL (2013). Genome-wide analysis reveals TET- and TDG-dependent 5-methylcytosine oxidation dynamics.. Cell.

[r60] Smeester L, Rager JE, Bailey KA, Guan X, Smith N, Garcia-Vargas G (2011). Epigenetic changes in individuals with arsenicosis.. Chem Res Toxicol.

[r61] Song CX, Szulwach KE, Dai Q, Fu Y, Mao SQ, Lin L (2013). Genome-wide profiling of 5-formylcytosine reveals its roles in epigenetic priming.. Cell.

[r62] Steinmaus C, Bates MN, Yuan Y, Kalman D, Atallah R, Rey OA (2006). Arsenic methylation and bladder cancer risk in case-control studies in Argentina and the United States.. J Occup Environ Med.

[r63] Steinmaus CM, Yuan Y, Smith AH (2005). The temporal stability of arsenic concentrations in well water in western Nevada.. Environ Res.

[r64] Stuart EA, Hanna DB (2013). Should epidemiologists be more sensitive to design sensitivity?. Epidemiology.

[r65] Su PF Hu YJ Ho IC Cheng YM Lee TC 2006 Distinct gene expression profiles in immortalized human urothelial cells exposed to inorganic arsenite and its methylated trivalent metabolites. Environ Health Perspect 114 394 403; 10.1289/ehp.8174 16507463 PMC1392234

[r66] Tahiliani M, Koh KP, Shen Y, Pastor WA, Bandukwala H, Brudno Y (2009). Conversion of 5-methylcytosine to 5-hydroxymethylcytosine in mammalian DNA by MLL partner TET1.. Science.

[r67] Tajuddin SM Amaral AF Fernández AF Rodríguez-Rodero S Rodríguez RM Moore LE 2013 Genetic and non-genetic predictors of LINE-1 methylation in leukocyte DNA. Environ Health Perspect 121 650 656; 10.1289/ehp.1206068 23552396 PMC3672919

[r68] Takiguchi M, Achanzar WE, Qu W, Li G, Waalkes MP (2003). Effects of cadmium on DNA-(cytosine-5) methyltransferase activity and DNA methylation status during cadmium-induced cellular transformation.. Exp Cell Res.

[r69] Tellez-Plaza M Gribble MO Voruganti VS Francesconi KA Goessler W Umans JG 2013 Heritability and preliminary genome-wide linkage analysis of arsenic metabolites in urine. Environ Health Perspect 121 345 351; 10.1289/ehp.1205305 23322787 PMC3621197

[r70] Terry MB, Delgado-Cruzata L, Vin-Raviv N, Wu HC, Santella RM (2011). DNA methylation in white blood cells: association with risk factors in epidemiologic studies.. Epigenetics.

[r71] Vahter M (2000). Genetic polymorphism in the biotransformation of inorganic arsenic and its role in toxicity.. Toxicol Lett.

[r72] Valko M, Morris H, Cronin MT (2005). Metals, toxicity and oxidative stress.. Curr Med Chem.

[r73] Wu MM, Chiou HY, Hsueh YM, Hong CT, Su CL, Chang SF (2006). Effect of plasma homocysteine level and urinary monomethylarsonic acid on the risk of arsenic-associated carotid atherosclerosis.. Toxicol Appl Pharmacol.

[r74] Xu J, Lee ET, Peterson LE, Devereux RB, Rhoades ER, Umans JG (2012). Differences in risk factors for coronary heart disease among diabetic and nondiabetic individuals from a population with high rates of diabetes: the Strong Heart Study.. J Clin Endocrinol Metab.

[r75] Xu W, Yang H, Liu Y, Yang Y, Wang P, Kim SH (2011). Oncometabolite 2-hydroxyglutarate is a competitive inhibitor of α-ketoglutarate-dependent dioxygenases.. Cancer Cell.

[r76] Yu RC, Hsu KH, Chen CJ, Froines JR (2000). Arsenic methylation capacity and skin cancer.. Cancer Epidemiol Biomarkers Prev.

[r77] Zubizarreta JR, Cerda M, Rosenbaum PR (2013). Effect of the 2010 Chilean earthquake on posttraumatic stress: reducing sensitivity to unmeasured bias through study design.. Epidemiology.

