# Supplementary material for: Association of Global DNA Methylation and Global DNA Hydroxymethylation with Metals and Other Exposures in Human Blood DNA Samples
Source: Environ Health Perspect. 2014 Apr 25;122(9):946–54. doi: 10.1289/ehp.1306674 (PMC4154208; doi:10.1289/ehp.1306674)
Supplement: (1.3 MB) PDF [file ehp.1306674.s001.pdf]

Supplemental Material

Association of Global DNA Methylation and Global DNA Hydroxymethylation with Metals and other Exposures in Human Blood DNA Samples

Maria Tellez-Plaza, Wan-yee Tang, Yan Shang, Jason G. Umans, Kevin A. Francesconi, Walter Goessler, Marta Ledesma, Montserrat Leon, Martin Laclaustra, Jonathan Pollak, Eliseo Guallar, Shelley A. Cole, M. Dani Fallin, and Ana Navas-Acien

Table of Contents **Page**

Table S1. Intra-assay coefficient of variation (CV) and intra-class correlation (ICC) for the technical replicates in the Strong Heart Study (SHS) (Lee et al. 1990; North et al. 2003) and Aragon Workers Health Study (AWHS) (Casasnovas et al. 2012) samples **2**

Table S2. Distribution of white blood cell counts in the SHS (Lee et al. 1990; North et al. 2003) blood samples (visit 3) **3**

Table S3. Median (interquartile range) of global DNA methylation (% of 5-mC) and global DNA hydroxymethylation (% of 5-hmC) in the SHS (Lee et al. 1990; North et al. 2003) in 1989-1991 (visit 1) and 1993-1995 (visit 3)[a] by participant characteristics **4**

Figure S1. Relationship of global DNA methylation (% 5-mC) and participant characteristics in the SHS (Lee et al. 1990; North et al. 2003) in 1989-1991 (visit 1) and 1998-1999 (visit 3) **6**

Figure S2. Relationship of global DNA hydroxymethylation (% 5-hmC) and participant characteristics in the SHS (Lee et al. 1990; North et al. 2003) in 1989-1991 (visit 1) and 1998-1999 (visit 3) **7**

Figure S3. Relationship of global DNA methylation (% 5-mC) and global DNA hydroxymethylation (% 5-hmC,) in blood collected in 2010, Aragon Workers Health Study (Casasnovas et al. 2012) **8**

References **9**

Table S1. Intra-assay coefficient of variation (CV) and intra-class correlation (ICC) for the technical replicates in the Strong Heart Study (SHS) (Lee et al. 1990; North et al. 2003) and Aragon Workers Health Study (AWHS) (Casasnovas et al. 2012) samples.

**Visit**	**5-mC assay: replicates**	**5-mC assay: median CV (IQR)**	**5-mC assay: ICC**	**5-hmC assay: replicates**	**5-hmC assay: median CV (IQR)**	**5-hmC assay: ICC**
**Strong Heart Study**						
Visit 1, N=48	3	0.25% (0.10%, 0.51%)	0.9999	3	0.37% (0.20%, 0.69%)	0.9993
Visit 3, N=48	3	0.26% (0.08%, 0.58%)	0.9998	3	0.31% (0.16%, 1.35%)	0.9985*
**Aragon Workers Health Study**						
N=48	3	3.14% (3.05%, 3.20%)	0.860	2	2.80% (1.40%, 4.30%)	0.614

*One individual with and outlier observance reading equal to zero excluded.

Table S2. Distribution of white blood cell counts in the SHS (Lee et al. 1990; North et al. 2003) blood samples (visit 3).

Counts	N	Median (IQR)
Total white blood cells (units x 10^3/uL)	44	6.25 (5.40, 7.35)
Neutrophils (units x 10^3/uL)	44	3.65 (3.25, 4.58)
Lymphocyte (units x 10^3/uL)	44	1.65 (1.45, 2.07)
Monocyte (units x 10^3/uL)	44	0.36 (0.28, 0.49)
Eosinophils (units x 10^3/uL)	39	0.20 (0.13, 0.29)
Basophil (units x 10^3/uL)	44	0.05 (0.03, 0.12)

Information on blood cell counts in visit 3 was only available for 44 participants, among them only 39 participants had information on eosinophil count.

Table S3. Median (interquartile range) of global DNA methylation (% of 5-mC) and global DNA hydroxymethylation (% of 5-hmC) in the SHS (Lee et al. 1990; North et al. 2003) in 1989-1991 (visit 1) and 1993-1995 (visit 3)[a] by participant characteristics.

Characteristics	N	Methylation, Visit 1	Methylation, Visit 3	Hydroxymethylation, Visit 1	Hydroxymethylation, Visit 3
**Overall**	48	0.32 (0.15, 0.58)	0.32 (0.13, 0.55)	0.12 (0.07, 0.17)	0.15 (0.09, 0.25)
**Age, years**					
<54	24	0.34 (0.23, 0.60)	0.36 (0.27, 0.60)	0.13 (0.08, 0.19)	0.15 (0.09, 0.28)
≥54	24	0.31 (0.12, 0.54)	0.22 (0.10, 0.45)	0.12 (0.05, 0.15)	0.13 (0.07, 0.19)
**Sex**					
Male	15	0.35 (0.12, 0.51)	0.33 (0.20, 0.67)	0.12 (0.07, 0.14)	0.13 (0.09, 0.19)
Female	33	0.32 (0.17, 0.68)	0.32 (0.11, 0.46)	0.13 (0.07, 0.18)	0.16 (0.09, 0.28)
**Education, years**					
≥12	29	0.28 (0.13, 0.53)	0.32 (0.16, 0.42)	0.13 (0.08, 0.19)	0.12 (0.08, 0.19)
<12	19	0.39 (0.24, 0.61)	0.35 (0.10, 0.58)	0.09 (0.05, 0.15)	0.17 (0.11, 0.23)
**BMI, kg/m**[b]					
<30	20	0.26 (0.13, 0.49)	0.21 (0.09, 0.37)	0.12 (0.08, 0.17)	0.14 (0.09, 0.22)
≥30	27	0.35 (0.18, 0.62)	0.37 (0.24, 0.62)	0.13 (0.07, 0.18)	0.16 (0.09, 0.26)
**Waist circumference, cm**[b]					
<Sex-specific median	20	0.34 (0.17, 0.60)	0.27 (0.11, 0.55)	0.12 (0.07, 0.15)	0.16 (0.08, 0.26)
≥Sex-specific median	26	0.32 (0.13, 0.58)	0.33 (0.16, 0.57)	0.13 (0.08, 0.18)	0.14 (0.09, 0.19)
**% Body fat**[b]					
<Sex-specific median	23	0.32 (0.16, 0.61)	0.28 (0.10, 0.47)	0.10 (0.05, 0.16)	0.13 (0.06, 0.23)
≥Sex-specific median	24	0.31 (0.13, 0.51)	0.35 (0.20, 0.69)	0.13 (0.09, 0.18)	0.16 (0.10, 0.27)
**Smoking**					
Never	20	0.32 (0.18, 0.58)	0.41 (0.26, 0.63)	0.12 (0.08, 0.15)	0.17 (0.12, 0.26)
Ever	28	0.33 (0.12, 0.58)	0.25 (0.13, 0.38)	0.12 (0.06, 0.17)	0.12 (0.07, 0.23)
**Alcohol status**					
Never	8	0.39 (0.12, 0.72)	0.36 (0.10, 0.54)	0.16 (0.08, 0.21)	0.13 (0.10, 0.16)
Ever	40	0.32 (0.16, 0.56)	0.32 (0.15, 0.58)	0.12 (0.07, 0.16)	0.15 (0.08, 0.27)
**Urine arsenic μg/g**[c]					
<7.2	24	0.33 (0.13, 0.58)	0.34 (0.23, 0.62)	0.13 (0.06, 0.23)	0.16 (0.09, 0.25)
≥14.0	24	0.32 (0.18, 0.57)	0.30 (0.09, 0.42)	0.12 (0.08, 0.15)	0.14 (0.07, 0.22)
**% iAs**[b]					
< 8.5	23	0.35 (0.21, 0.63)	0.33 (0.15, 0.50)	0.14 (0.09, 0.18)	0.17 (0.11, 0.28)
≥ 8.5	23	0.32 (0.14, 0.57)	0.28 (0.10, 0.58)	0.10 (0.04, 0.17)	0.11 (0.08, 0.19)
**% MAA**[b]					
< 14.4	23	0.39 (0.20, 0.68)	0.37 (0.17, 0.59)	0.15 (0.11, 0.19)	0.16 (0.11, 0.21)
≥ 14.4	23	0.31 (0.15, 0.50)	0.25 (0.11, 0.38)	0.08 (0.04, 0.14)	0.11 (0.06, 0.26)
**% DMA**[b]					
< 78.3	23	0.28 (0.14, 0.45)	0.25 (0.10, 0.39)	0.10 (0.05, 0.15)	0.11 (0.07, 0.24)
≥ 78.3	23	0.39 (0.28, 0.73)	0.37 (0.19, 0.56)	0.14 (0.09, 0.19)	0.16 (0.11, 0.23)
**Cadmium, μg/g**					
<0.87	24	0.25 (0.12, 0.47)	0.30 (0.16, 0.55)	0.11 (0.06, 0.16)	0.13 (0.08, 0.21)
≥0.87	24	0.39 (0.29, 0.70)	0.35 (0.13, 0.55)	0.13 (0.08, 0.17)	0.16 (0.09, 0.26)
**Antimony, μg/g**					
<0.27	24	0.29 (0.16, 0.40)	0.24 (0.10, 0.38)	0.13 (0.09, 0.15)	0.15 (0.08, 0.21)
≥0.27	24	0.43 (0.13, 0.68)	0.38 (0.24, 0.59)	0.10 (0.05, 0.21)	0.15 (0.09, 0.25)
**Tungsten, μg/g**					
<0.13	24	0.32 (0.12, 0.44)	0.32 (0.19, 0.48)	0.12 (0.05, 0.15)	0.14 (0.09, 0.18)
≥0.13	24	0.36 (0.17, 0.79)	0.31 (0.13, 0.59)	0.13 (0.08, 0.18)	0.20 (0.09, 0.28)

Abbreviations: IQR, interquartile range; %iAs, Percentage of inorganic arsenic over the sum of inorganic and methylated arsenic species; %MMA, percentage of monomethylarsonate over the sum of inorganic and methylated arsenic species; %DMA, percentage of dimethylarsinate over the sum of inorganic and methylated arsenic species. [a]Levels of 5-mC or 5-hmC in DNA of all biological samples were measured as the amount of methylated or hydroxymethylated cystosines relative to the cytosine genomic content (%). The median (interquartile range) level for global DNA methylation and global DNA hydroxymethylation, relative to the total DNA content was 0.13 (0.06, 0.24) % of 5-mC and 0.05 (0.03, 0.07) % of 5-hmC in visit 1 and 0.13 (0.05, 0.23) % of 5-mC and 0.06 (0.04, 0.09) % of 5-hmC in visit 3. [b]For BMI, Waist circumference, %body fat, and arsenic species the total size do not sum 48 because of missing data. [c]Sum of inorganic and methylated arsenic species in urine.

Figure S1. Relationship of global DNA methylation (% 5-mC) and participant characteristics in the SHS (Lee et al. 1990; North et al. 2003) in 1989-1991 (visit 1) and 1998-1999 (visit 3).

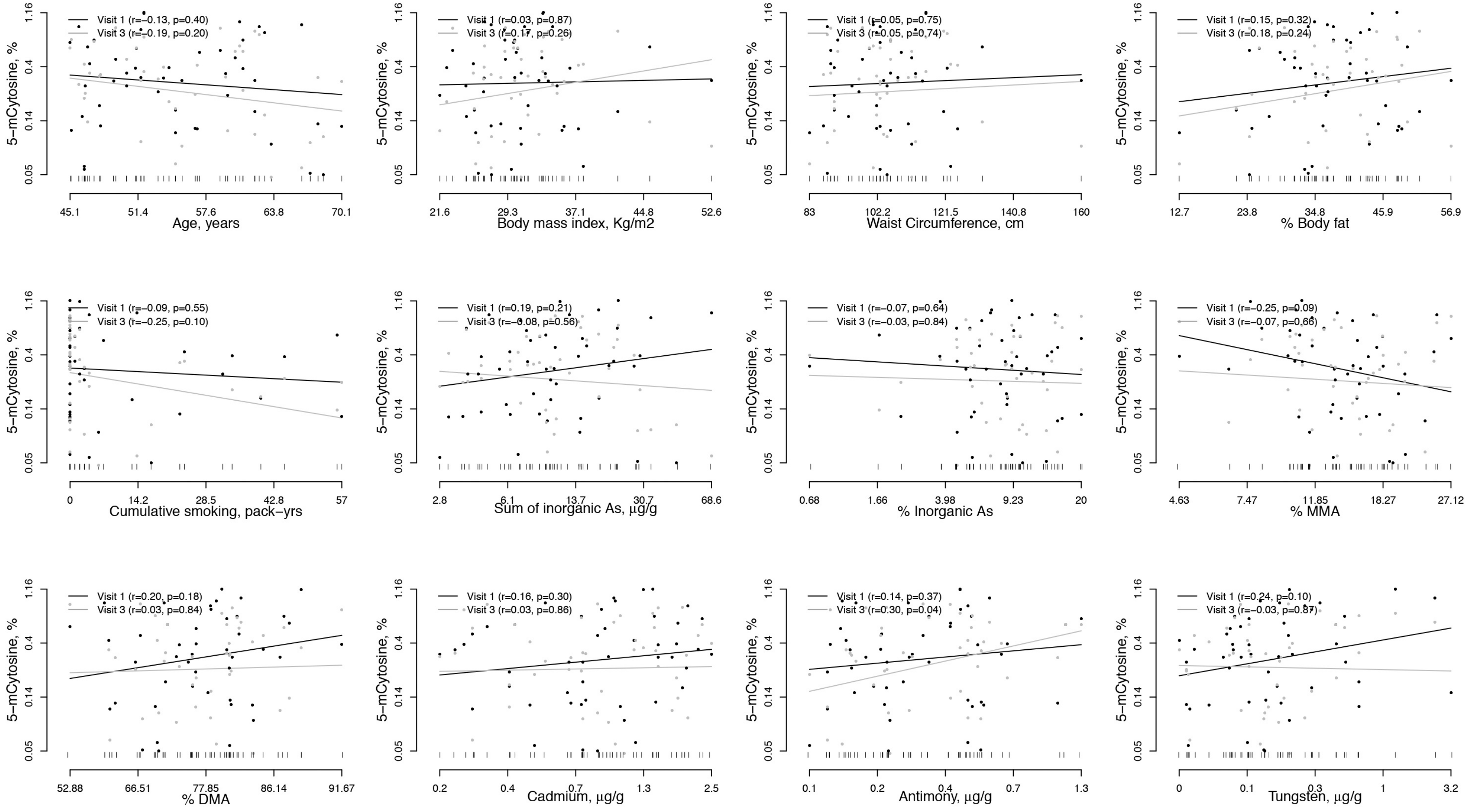

Figure S2. Relationship of globa DNA hydroxymethylation (% 5-hmC) and participant characteristics in the SHS (Lee et al. 1990; North et al. 2003) in 1989-1991 (visit 1) and 1998-1999 (visit 3).

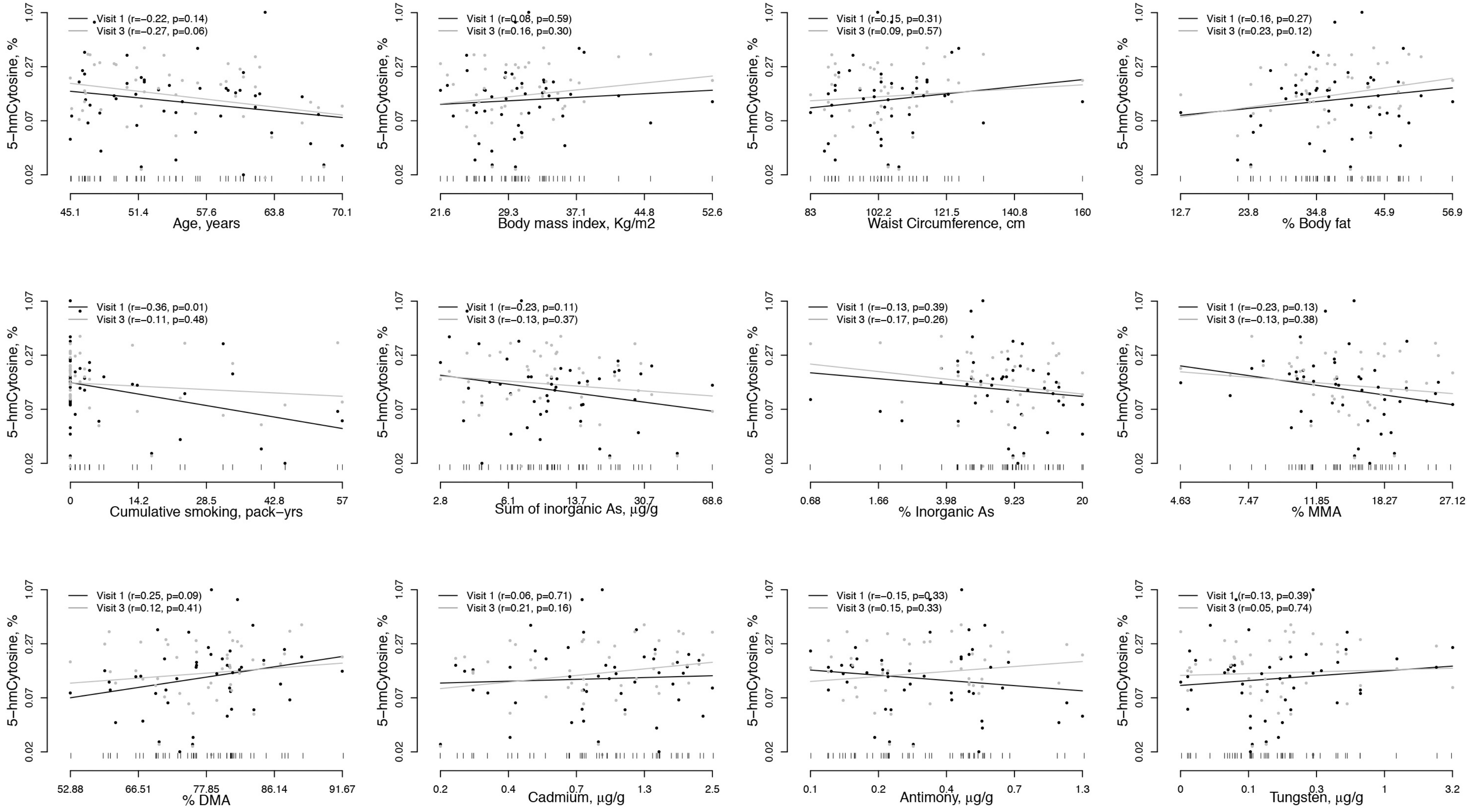

Figure S3. Relationship of global DNA methylation (% 5-mC) and global DNA hydroxymethylation (% 5-hmC,) in blood collected in 2010, Aragon Workers Health Study (Casasnovas et al. 2012).

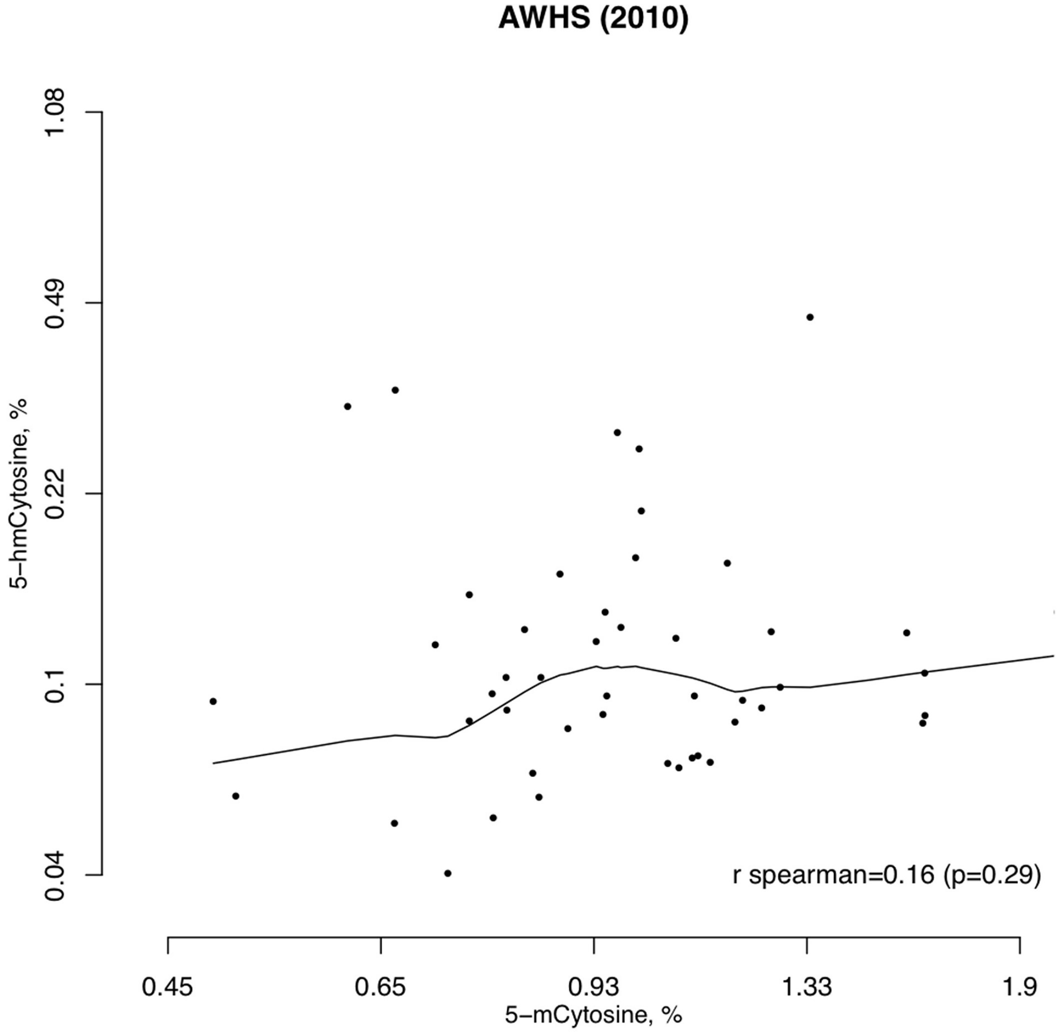

References

Casasnovas JA, Alcaide V, Civeira F, Guallar E, Ibanez B, Borreguero JJ, et al. 2012. Aragon Workers' Health Study--design and cohort description. BMC cardiovascular disorders 12:45.

Lee ET, Welty TK, Fabsitz R, Cowan LD, Le NA, Oopik AJ, et al. 1990. The Strong Heart Study. A study of cardiovascular disease in American Indians: Design and methods. American journal of epidemiology 132:1141-1155.

North KE, Howard BV, Welty TK, Best LG, Lee ET, Yeh JL, et al. 2003. Genetic and environmental contributions to cardiovascular disease risk in American Indians: The Strong Heart Family Study. American journal of epidemiology 157:303-314.
